# *Pseudotrichonympha leei*, *Pseudotrichonympha lifesoni*, and *Pseudotrichonympha pearti*, new species of parabasalian flagellates and the description of a rotating subcellular structure

**DOI:** 10.1038/s41598-017-16259-8

**Published:** 2017-11-27

**Authors:** Javier del Campo, Erick R. James, Yoshihisa Hirakawa, Rebecca Fiorito, Martin Kolisko, Nicholas A. T. Irwin, Varsha Mathur, Vittorio Boscaro, Elisabeth Hehenberger, Anna Karnkowska, Rudolf H. Scheffrahn, Patrick J. Keeling

**Affiliations:** 1 Department of Botany, University of British Columbia, Vancouver, British Columbia Canada; 2 Department of Marine Biology and Oceanography, Institut de Ciències del Mar - CSIC, Barcelona, Catalonia Spain; 3 Institute of Parasitology, Biology Centre, Czech Academy of Sciences, České Budějovice, Czech Republic; 4 Department of Molecular Phylogenetics and Evolution, Faculty of Biology and Biological and Chemical Research Centre, University of Warsaw, Warsaw, Poland; 5 Fort Lauderdale Research & Education Center, University of Florida, Davie, Florida USA

## Abstract

*Pseudotrichonympha* is a large and structurally complex genus of parabasalian protists that play a key role in the digestion of lignocellulose in the termite hindgut. Like many termite symbionts, it has a conspicuous body plan that makes genus-level identification relatively easy, but species-level diversity of *Pseudotrichonympha* is understudied. Molecular surveys have suggested the diversity is much greater than the current number of described species, and that many “species” described in multiple hosts are in fact different, but gene sequences from formally described species remain a rarity. Here we describe three new species from *Coptotermes* and *Prorhinotermes* hosts, including small subunit ribosomal RNA (SSU rRNA) sequences from single cells. Based on host identification by morphology and DNA barcoding, as well as the morphology and phylogenetic position of each symbiont, all three represent new *Pseudotrichonympha* species: *P. leei, P. lifesoni*, and *P. pearti*. *Pseudotrichonympha leei* and *P. lifesoni*, both from *Coptotermes*, are closely related to other *Coptotermes* symbionts including the type species, *P. hertwigi*. *Pseudotrichonympha pearti* is the outlier of the trio, more distantly related to *P. leei* and *P. lifesoni* than they are to one another, and contains unique features, including an unusual rotating intracellular structure of unknown function.

## Introduction

*Pseudotrichonympha* is a genus of parabasalian protists found exclusively in the hindguts of lower termites, in particular rhinotermitids, where they play a key role in a well-studied symbiotic system in which the microbial community degrades the lignocellulose that makes up most of the animal’s diet^1^. The ancestral parabasalian body plan was probably a relatively simple cell, characterized by the presence of a hydrogenosome, an anaerobic metabolic organelle derived from the mitochondrion, and akaryomastigont system comprising a nucleus, four flagella, and other conserved cytoskeletal elements^2^. But the morphology of parabasalians diversified greatly within the context of symbiosis with insects: cells expanded in size, and in some lineages cytoskeletal elements were replicated to form patterns both so grand and complex that they were considered to form a lineage, the so-called hypermastigotes^3^. Molecular phylogeny and morphology have now shown this elaboration of form actually happened independently in several parabasalian subgroups^4^. *Pseudotrichonympha* is a member of one such group, the Trichonymphida, which are characterized by very large cells with a single nucleus that are covered by thousands or tens of thousands of flagella, typically organized in longitudinal rows that extend over most of the cell body and emerge from an anterior organizing centre beneath an apical cap. Other hypermastigotes can be found in the class Cristamonadea, which typically have multiple karyomastigonts rather than a single nucleus (with exceptions such as *Deltotrichonympha* and *Kofoidia*), and in the class Spirotrichonymphea, where flagellar bands are organized in a spiral array consisting of multiple right-handed helices.

The overall body plan of *Pseudotrichonympha* matches the main characteristics of Trichonymphida as a whole, and is specifically distinguished by a robust apical cap, a sub-apical rostrum with an anterior band of short flagella and a posterior band of distinctively long flagella, and a main body that is typically elongated and covered with shorter flagella organized in longitudinal rows (which may have a slight spiral on close inspection). *Pseudotrichonympha* occurs in many different termite host species, but its diversity and taxonomy are both understudied, and a long history of mistaken identities has led to too much contradictions and confusion in its classification. The genus was first described over a century ago, but was initially and erroneously interpreted as one of the ‘sexes’ of *Trichonympha* ^5^. This was corrected by the creation of the new genus, with the type species *Pseudotrichonympha hertwigi* ^6^. The type species has since been re-examined by light and electron microscopy and its small subunit rRNA gene sequences (SSU rRNA) characterized for phylogenetic analysis^7^. But most of the diversity of *Pseudotrichonympha* remains unexamined. They are known to be common, perhaps ubiquitous^8^, in the rhinotermitids, a widely-distributed and speciose family of termites, but only about a dozen species have been formally described^7^. Their diversity is also evident from molecular surveys of termite hindgut communities, which have yielded a diverse clade of environmental sequences that are inferred to be from *Pseudotrichonympha*, but currently sequences from organisms lacking formal taxonomic description outnumber sequences from described species by a ratio of five to one^7^. Here, we describe three new species of *Pseudotrichonympha* from barcoded *Coptotermes* and *Prorhinotermes* hosts from North America and Australia, using single cell isolation to provide molecular data from the SSU rRNA for phylogenetic analysis so as to further fill the spaces between identified species and environmental sequences in the *Pseudotrichonympha* tree.

## Results and Discussion

### Host collection and identification

**Figure 1:**
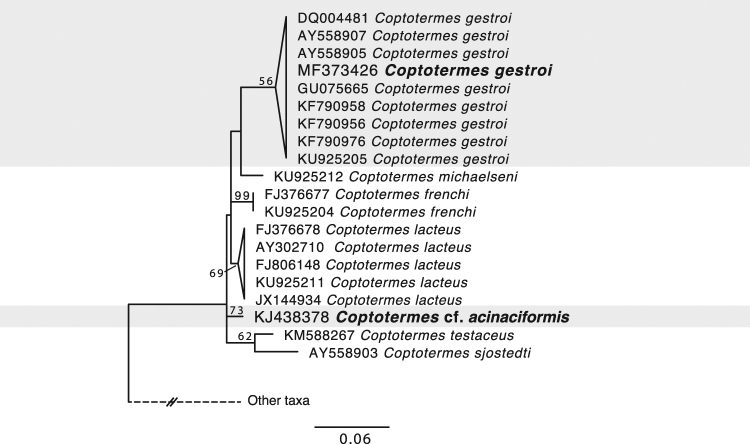
Maximum likelihood phylogeny of host barcode sequences (mitochondrial LSU rRNA) from *Coptotermes*. All available *Coptotermes* barcodes are included. The new specimen from Florida is confirmed to correspond to *C. gestroi*, while the new specimen from Australia is a species for which no barcode is currently available. Based on the available barcodes to which it does not correspond and the diversity of *Coptotermes* from this region of Queensland, we infer this is most likely a specimen of *C. acinaciformis*, and therefore refer to this as *Coptotermes* cf. *acinaciformis*. Conspecific clades are collapsed and represented by triangles. Numbers at nodes correspond to ML bootstrap support over 50% (values for nodes with lower support are not shown for clarity), and the scale bar represents a distance of 0.06 substitutions per site. The complete tree with outgroups can be seen in Suppl. Figure 1.

*Prorhinotermes simplex* and *Coptotermes gestroi* were collected in Fort Lauderdale, Florida, USA, and their morphological identification was confirmed by DNA barcoding (accessions JX975355 and MF373426, respectively). A second species of *Coptotermes* was collected in Mount Glorious, Queensland, Australia. It was identified to the genus level by morphology in the field. Ethanol-preserved samples were returned to the lab for DNA barcoding using the mitochondrial large subunit rRNA gene (accession KJ438378), which allowed for comparisons with two of the three species of *Coptotermes* known from the area. *Coptotermes frenchi* barcodes shared on average 95.5% identity with the Australian barcode and showed no close affinity in the phylogenetic tree (Fig. 1, Suppl. Figure 1). The identity shared with *Coptotermes lacteus* barcodes was on average 96.7%, and in phylogenetic trees the Australian barcode branched closer to *C. lacteus*, but did not branch within the clade of *C. lacteus* barcode isolates (which share 99–100% identity). Based on this, we conclude that the collection corresponds to neither *C. lacteus* nor *C. frenchi*. The only other *Coptotermes* known in this region is *Coptotermes acinaciformis*, but unfortunately there is no barcoded vouchered specimen of this species (*Coptotermes* are notorious difficult to identify). Therefore, we conclude that the probable identity of our isolate is *C. acinaciformis*, but it could also be a presently unknown species. We will henceforth refer to this specimen as *Coptotermes* cf. *acinaciformis*.

### Morphology of new *Pseudotrichonympha* species

**Figure 2:**
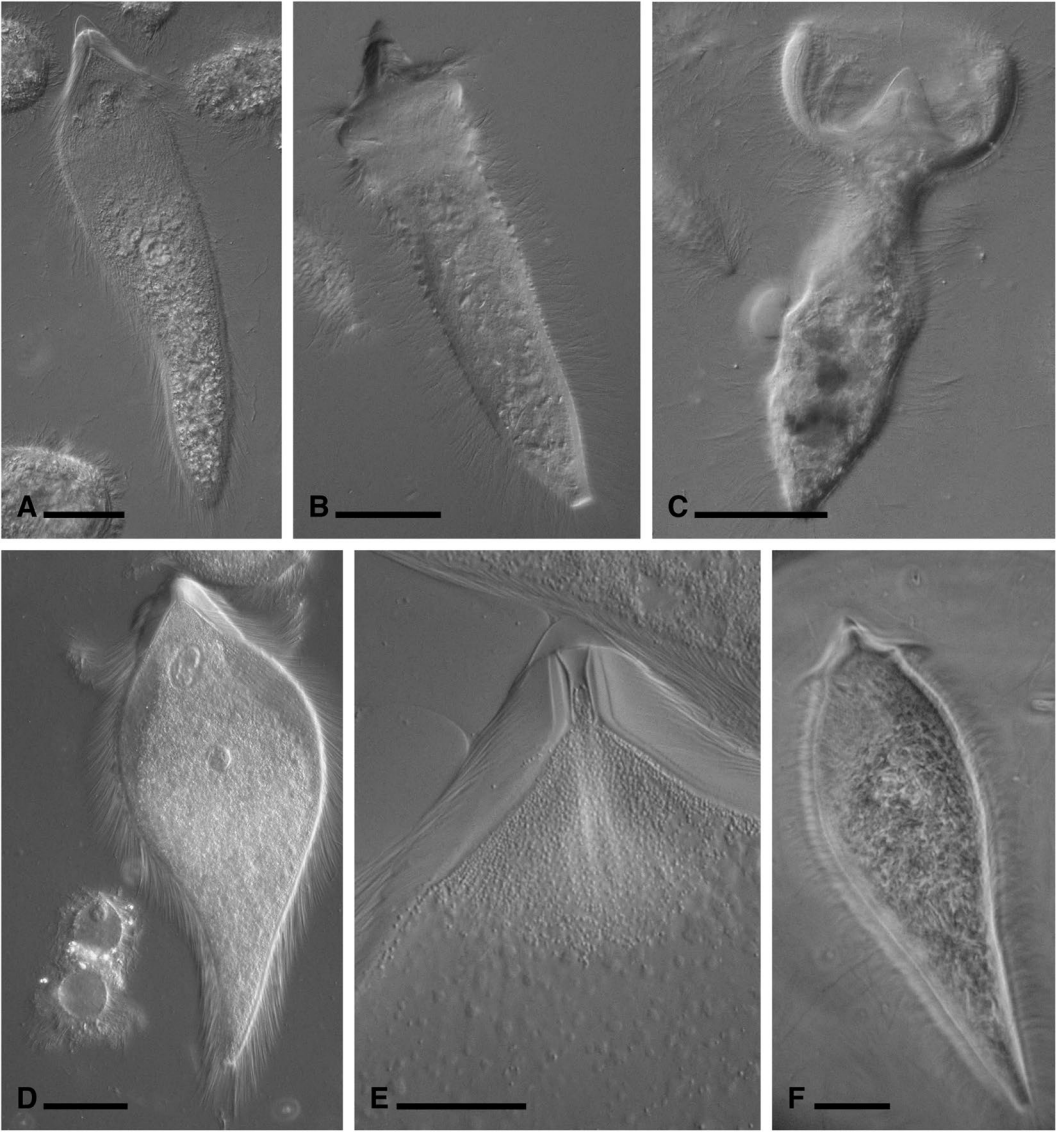
Light micrographs of new *Pseudotrichonympha* specimens. (**A**–**C**) Differential interference contrast images of *Pseudotrichonympha leei* from *Coptotermes gestroi* showing the overall body shape, with the long posterior rostral flagella emerging below the pointed apical cap, and shorter post-rostral flagella (**A**). (**B**,**C**) Show the commonly observed robust collar (**B**) and cup-shaped anterior of stressed cells (**C**). (**D**,**E**) Differential interference contrast images of *Pseudotrichonympha pearti* from *Prorhinotermes simplex* showing the overall body shape, single nucleus, and detail of the rostral region, including the apical cap, rostral tube, and long posterior rostral flagella. (**F**) Phase contrast images (taken in the field) of *Pseudotrichonympha lifesoni* from *Coptotermes* cf. *acinaciformis* showing the basic body shape and size and the single central nucleus. Bars represent 50 µm.

In all three termite species we observed and photographed cells matching the description of *Pseudotrichonympha* (Fig. 2). Specifically, all three symbionts were characterized by an apical cap with an adjacent apical band of short rostral flagella, a distinctive posterior band of very long rostral flagella, and intermediate-length flagella covering the remainder of the post-rostral body. Cells contained a single nucleus central in the sub-rostral area (e.g. see Fig. 2E). The *Pseudotrichonympha* from *Coptotermes cf*. *acinaciformis* (Fig. 2F) was very large (length average: 392 µm, length range: 332–448 µm, width average: 100 µm,width range: 94–108, n = 5) and had a wide ovoid shape and a sharply tapering posterior tip. The *Pseudotrichonympha* from *C. gestroi* (Fig. 2A–C) had a more slender body (length average: 303 µm, length range: 280–330 µm, width average: 62 µm,width range: 56–71 µm, n = 5), and was distinguished by the tendency to possess a robust sub-apical shoulder region that frequently had the greatest diameter of the body (Fig. 2B). In stressed cells (e.g. during isolation), this region expanded greatly and produced a distinctive cup-shaped cell with the apex at the centre of the cup (Fig. 2C). The *Pseudotrichonympha* from *P. simplex* (Fig. 2D,E) tended to have a wide body around the midline (length average: 262 µm, length range: 200–316 µm, width average: 122 µm, width range: 89–180 µm, n = 12), and was most obviously defined by the presence of a strange intracellular body we dubbed the “rotatosome”.

**Figure 3:**
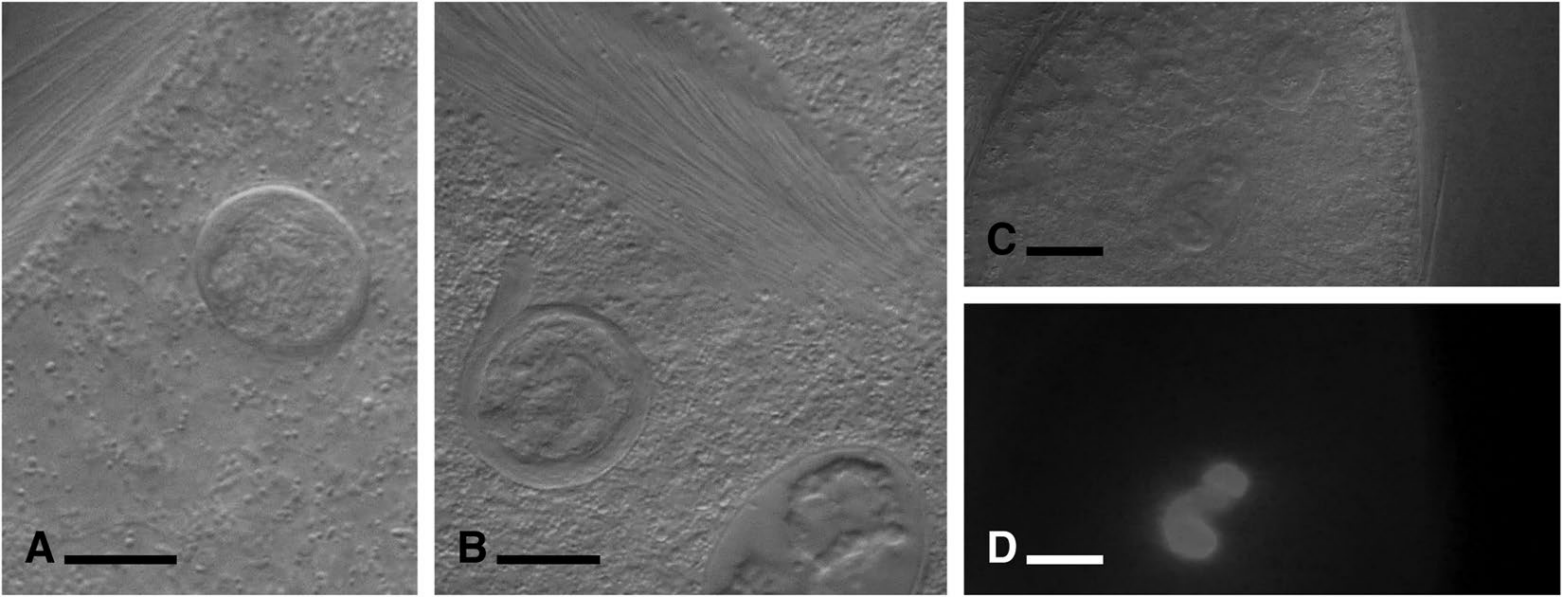
Details of the “rotatosome” from *Pseudotrichonympha pearti*. Differential interference contrast details of the rotating structure in the *P. pearti* cytoplasm (**A**,**B**), with (**B**) showing the projection into the cytoplasm (this can also be seen in the Supplemental video, which also shows how the structure rotates). (**C** and **D**) show a rotatosome next to the *P. pearti* nucleus in fixed and Hoechst stained cells. In C (the DIC image) both structures are visible, but in D (Hoechst stain fluorescence) only the nucleus can be seen to fluoresce, showing the rotatosome does not contain DNA. Bars represent 10 µm.

The rotatosome (see Fig. 3, and Supplemental video files) is a spherical body on average 13 µm in diameter (n = 3) that was only ever observed in a single copy per cell. It could only be observed in some focal planes, so whether it is in all cells or not is not known. It continuously rotated, causing visible turbulence in the cytoplasm around it. The lumen of the rotatosome was not uniform. At certain observational planes the rotatosome could be seen to include a rod 6-7 µm in length that emerged from one side of the circle (which side depended on the angle from which it was viewed), was stationary with respect to the cytoplasm, and in which the material appears to stream towards the outermost layer of the sphere, where it can then be seen to turn and turn again in the direction of the rotation. We examined the possibility that the rotatosome is an endosymbiont living within *Pseudotrichonympha* by Hoechst staining. In fixed and stained cells, the nucleus consistently showed a strong signal for the presence of DNA (in the pattern expected for a parabasalian, which have condensed interphase chromosomes), but the rotatosome retained no detectible stain (Fig. 3C,D). Similarly, we manually broke three *Pseudotrichonympha* cells open under the microscope to see if a rotatosome could be observed swimming freely, but the body consistently disappeared when cells were disrupted (not shown). A spherical structure was previously observed, in 1923, in the cytoplasm of a species identified as *P. sphaerophora* from a termite identified as *Rhinotermes nasutus* in British Guiana^9^. In this case, the sphere was reported to be larger (25 µm), consisting of a non-staining centre covered by a lighter layer in a concentric organization. The structured was reported from a fixed and stained sample, so no rotation was observed, and it was hypothesized at the time to function as a stercoma for storing excretory material. Based on the difference in size, lack of detail in the 1923 description, and the fact this is apparently not the same species of host or symbiont, or the same collection location, it would be premature to conclude these are the same structure. Overall, in either case, many questions remain about the function and the form of the rotatosome that will hopefully be resolved in the fullness of time, but it appears to be a sub-cellular structure rather than an endosymbiont.

### Molecular phylogeny

**Figure 4:**
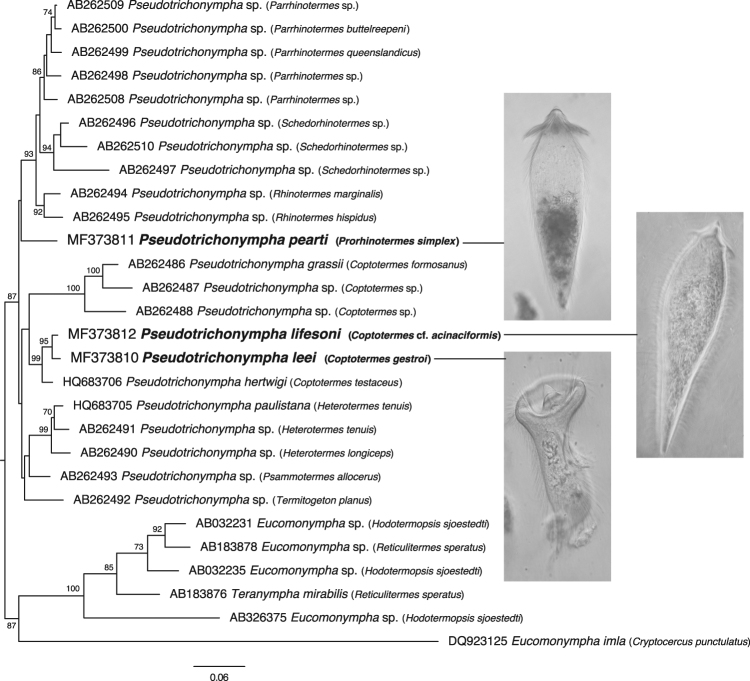
Maximum likelihood (ML) phylogeny of SSU rRNA genes from *Pseudotrichonympha*, showing the relationships between *Pseudotrichonympha leei*, *Pseudotrichonympha lifesoni*, and *Pseudotrichonympha pearti*, to other members of the genus. The tree is rooted with the closest known relatives to *Pseudotrichonympha*, *Teranympha* and *Eucomonypha*. Numbers at nodes correspond to ML bootstrap support over 70% (values for nodes with lower support are not shown for clarity), and the scale bar represents a distance of 0.06 substitutions per site. Taxon names include GenBank accession numbers and the name of the termite host, where known. Most of the diversity on the phylogeny is represented by sequences from undescribed species, many from unidentified hosts, emphasizing our dearth of knowledge about the diversity of this lineage.

To determine how these *Pseudotrichonympha* species relate to other members of the genus, we sequenced the SSU rRNA gene from single isolated cells and small pools of cells. In all cases, sequences from the symbionts of a given host were 98.5–99% identical and one representative sequence per taxon was used for phylogenetic analyses and submitted to GenBank under accessions MF373810 –MF373812. In phylogenetic trees, the *Pseudotrichonympha* symbionts from *Coptotermes* hosts branch within a single clade, albeit with no support (Fig. 4). However, within this clade are two well-supported subgroups, one of these consists of three sequences from Southeast Asian specimens of *Coptotermes* (and includes one known species, *P. grassii* from *C. formosanus*), and the other consists of *P. hertwigi* from its type host, *C. testaceus*, and the two *Coptotermes* symbionts described here. The *P. simplex* symbiont is excluded from this clade, but its position is otherwise unresolved and the real relation to the underlying tree of currently sampled *Pseudotrichonympha* sequences unclear (Fig. 4).

### Taxonomic considerations

All three specimens of *Pseudotrichonympha* characterized here represent new species. In the case of *C. gestroi*, flagellates have been reported^10^ but none, including members of the genus *Pseudotrichonympha*, have been formally described. In the case of *P. simplex*, several spirotrichonymphids and the smaller flagellate, *Cthulhu*, have been described^11^, but although *Pseudotrichonympha* has been reported to be present, it was never formally described^10–12^.

In the case of the Australian *Coptotermes* cf. *acinaciformis*, we can conclude the symbiont is undescribed without knowing the host species with absolute certainty. Of the three *Coptotermes* known to exist in the region, one (*C. frenchi*) has no described *Pseudotrichonympha*, and the other two (*C. lacteus* and *C. acinaciformis*) are both documented as containing “*Pseudotrichonympha hertwigi*”^10,13^. However, this host barcode excludes both *C. frenchi* and *C. lacteus*, and moreover the confusing history of *P. hertwigi* is inconsistent with the presence of this species in any of these hosts (reviewed in detail in ref.^7^). Since its original description and subsequent re-classificiation to a new genus^5,6,14^, several authors have assumed that *Pseudotrichonympha* observed in other *Coptotermes* hosts correspond to the same species, even in geographically distant Australian and Asian termites^10,12,13^. But this was invariably based only on light microscopy and limited numbers of distinguishable characters. The identity of the host species is also convoluted. The *Coptotermes* host of *P. hertwigi* was named *Coptotermes hartmanni* ^15^, but it lacked formal description and is now invalid^16^. Subsequent surveys of neotropical *Coptotermes* have shown all Brazilian specimens correspond to *Coptotermes testaceus*: this host has now been barcoded and the *P. hertwigi* SSU rRNA from this type host has been characterized^7^. This species does not exist in Australia, and neither the *Pseudotrichonympha* from the Australian *Coptotermes* nor the host barcodes match their Brazilian counterparts, leading to the conclusion that neither Australian *C. lacteus* nor *C. acinaciformis* (nor any other Australian termite) contain *P. hertwigi*, but rather contain undescribed species that have been misidentified. The same is also likely true for Asian termites concluded by light microscopy alone to harbour *P. hertwigi*. Based on this and the phylogenetic and morphological data, we propose three new species of *Pseudotrichonympha* as detailed below.

## Taxonomic Summary

### *Pseudotrichonympha leei* n. sp. del Campo and Keeling, 2017

urn:lsid:zoobank.org:act:33F52F74-954F-4F0F-9726-112C7D761266

**Type host**: *Coptotermes gestroi* (Isoptera, Rhinotermitidae: barcode MF373426).

**Type locality**: Ft. Lauderdale, Secrete Woods County Park, Florida, USA: lat. 26.08567, long. -80.18017.

**Host collection**: University of Florida termite collection, accession number FL3578. Collector R. H. Scheffrahn. Collected April 8, 2011.

**Description**: Parabasalian flagellate with morphological characteristics of the genus *Pseudotrichonympha*. Cells are on average 303 µm in length (range: 280–330 µm, n = 5) and 62 µm in width (range: 56–71 µm, n = 5). Cells are typically narrow and elongated, and can form a pronounced subapical collar, just posterior to the rostral flagella, which can, under stress, extend to give the cell a distinctive cup-shaped apex. Found in the hindgut of *Coptotermes gestroi*. Distinct SSU rRNA sequence (GenBank accession number MF373810).

**Holotype**: Specimen in Fig. 1A of the present publication.

**Gene sequence**: SSU rRNA accession number MF373810.

**Etymology**: Species name refers to the Geddy Lee, a musician who, with other members of Rush, have inspired an interest in natural history and science through art.

### *Pseudotrichonympha lifesoni* n. sp. del Campo and Keeling, 2017

urn:lsid:zoobank.org:act:C214D2FD-196A-4842-BCFF-9DA60F48B862

**Type host**: *Coptotermes* cf. *acinaciformis* (Isoptera, Rhinotermitidae: barcode KJ438378).

**Type locality**: Mount Glorious, 1780 Mt. Glorious Road, Queensland, Australia: lat. -27.33773, long. 152.77028.

**Host collection**: University of Florida termite collection, accession number AUS115. Collector P. J. Keeling. Collected Nov. 17, 2011.

**Description**: Parabasalian flagellate with morphological characteristics of the genus *Pseudotrichonympha*. Cells are on average 392 µm in length (range: 332–448 µm, n = 5) and 100 µm in width (range: 94–108 µm, n = 5). Cells are ovoid with a strongly elongated posterior tip and a clearly demarcated rostral flagellar zone. Found in the hindgut of *Coptotermes* cf. *acinaciformis* with a distinct barcode identification (KJ438378). Distinct SSU rRNA sequence (GenBank accession number MF373812).

**Holotype**: Specimen in Fig. 1F of the present publication.

**Gene sequence**: SSU rRNA accession number MF373812.

**Etymology**: Species name refers to the Alex Lifeson, a musician who, with other members of Rush, have inspired an interest in natural history and science through art.

### *Pseudotrichonympha pearti* n. sp. del Campo and Keeling, 2017

urn:lsid:zoobank.org:act:907E8D63-BA08-4B37-A36A-EDD7572D9A5E

**Type host**: *Prorhinotermes simplex* (Isoptera, Rhinotermitidae: barcode JX975355).

**Type locality**: Ft. Lauderdale, Secrete Woods County Park, Florida, USA: lat. 26.08567, long. -80.18017.

**Host collection**: University of Florida termite collection, accession number FL1563. Collector R. B. Maharajh. Collected Sept. 15, 2002.

**Description**: Parabasalian flagellate with morphological characteristics of the genus *Pseudotrichonympha*. Cells are on average 262 µm in length (range: 200–316 µm, n = 12) and 122 µm in width (range: 89–180 µm, n = 12). Cells contain a distinctive single, rotating spherical body, the rotatosome, with a diameter of approximately 13 µm. Found in the hindgut of *Prorhinotermes simplex*. Distinct SSU rRNA sequence (GenBank accession number MF373811)

**Holotype**: Specimen in Fig. 1D of the present publication.

**Gene sequence**: SSU rRNA accession number MF373811.

**Etymology**: Species name refers to the Neil Peart, a musician who, with other members of Rush, have inspired an interest in natural history and science through art.

## Methods

### Host termite collection and barcoding

*Prorhinotermes simplex* and *Coptotermes gestroi* were collected in Secret Woods County Park, Fort Lauderdale, Florida, USA, on September 15, 2002 and April 8, 2011, respectively. A *Coptotermes* that was identified morphologically to the genus level (probably *C. acinaciformis*) was collected at the Turkey Nest, Mount Glorious, Queensland, Australia, Nov. 17, 2011. All specimens were deposited in the University of Florida termite collection under accessions FL1563, FL3578, and AUS115, respectively. *Prorhinotermes simplex* and *Coptotermes gestroi* were maintained in conical tubes with wood from their habitats at room temperature in the laboratory. *Coptotermes* cf. *acinaciformis* was processed directly in the field. Termite identities were determined morphologically and by barcoding using the mitochondrial 16 S (LSU) ribosomal RNA gene amplified and sequenced using the primers LR-N-13398 5′-CGC CTG TTT ATC AAA AAC AT-3′^17^ and LR-J-13017 5′-TTA CGC TGT TAT CCC TAA-3′^18^. These were aligned with *Coptotermes* LSU termite barcodes from Genbank using using MAFFT v7.310^19^ (setting: –auto). Poorly aligned regions were automatically removed with trimAl v1.4 using a gap threshold of 0.3^20^ (settings: –gt 0.3 –st 0.001); columns with missing data at both ends were removed. For the termite phylogeny, maximum likelihood (ML) inference was carried out using RAxML v8.2.9 assuming the GTR + Γ substitution model with 1,000 random starting trees and statistical support obtained from 1,000 bootstrap replicates^21^. Barcodes were submitted to GenBank under accessions JX975355^11^, KJ438378^22^ and MF373426 (this study).

### Microscopy

Termites were dissected and hindgut contents were suspended in Trager’s medium U^23^. Symbionts from *C. gestroi* and *P. simplex* were observed on an Axioplan 2 compound microscope (Zeiss, Oberkochen, Germany) using differential interference contrast and documented with a 3CCD HD video camera XL H1S (Canon, Tokyo, Japan). Multiple cells of each new species were observed, filmed, and measured to estimate ranges of cell size and shape, but in all cases the size range was variable and many cells were observed to deviate from the norm, as these species are known to display significant plasticity. For this reason, we also carried out molecular characterization using single cell isolations of these symbionts, using an Axiovert 200 (Zeiss, Oberkochen, Germany) inverted microscope. Isolated cells were photographed with a MicroImager II (QImaging, Surrey, BC, Canada). For *Coptotermes* cf. *acinaciformis*, microscopy was carried out at the sampling location using a Swift Field Master phase contrast field microscope (Swift Optical Instruments, Schertz, TX) and imaging using a Canon G9 camera (Canon, Tokyo, Japan). Whether the “rotatosome” structure was an endosymbiotic cell was tested by puncturing individual cells with a small-diameter micro-pipette. We observed that with a certain amount of force the cell membrane could be disrupted without damaging comparably sized organelles, such as the nucleus.

For fluorescence microscopy, cells from *P. simplex* were fixed and stained in 2% paraformaldehyde in PBS buffer (pH 7.4) with 10 μg/mL Bisbenzimide H 33258 (Hoescht stain: Sigma, St Louis, MO) for 10 min. The cells were observed under an Axioplan2 fluorescent microscope and filmed with the Canon XL H1S. Bisbenzimide fluorescence were detected with the UV filter (excitation BP 365/12 nm, emission LP 397 nm).

### Single cell isolation, sequencing, and phylogenetic analysis

Individual *Pseudotrichonympha* cells were isolated by glass micropipette. Symbionts from *P. simplex* and *C. gestroi* were isolated in the lab using a Zeiss Axiovert, while individual *Pseudotrichonympha* cells from *Coptotermes* cf. *acinaciformis* were isolated directly in the field using the Swift Field Master. Representatives of this species were fixed in 95% ethanol with a view towards its preservation and shipped to the University of British Columbia for all subsequent molecular analyses. For all three species, single cells or small pools of cells were manually picked and rinsed three times under constant observation. In cases where the cell was so close to the edge of the cavity slide as to obscure the view, they were washed an additional time and inspected to confirm its state of integrity was not compromised. DNA was extracted from isolated cells using the Masterpure Complete DNA and RNA Purification Kit (Epicentre, Madison, WI, USA). SSU rRNA genes were amplified from purified DNA using the eukaryote specific primers PFI 5′-TGC GCT ACC TGG TTG ATC CTG CC-3′ and FAD4 5′-TGA TCC TTC TGC AGG TTC ACC TAC-3′. PCR conditions included a 3-minute denaturation at 95 °C followed by 30 cycles of 95 °C for 30 seconds, 55 °C for 30 seconds, and 72 °C for 1 minute 30 seconds, then an additional 7 minutes at 72 °C. Products were purified, cloned into the pCR2.1 vector using the TOPO-TA cloning kit (Invitrogen, Carlsbad, CA, USA), and sequenced on both strands with BigDye Terminator v 3.1 (Applied Biosystems, Carlsbad, CA, USA). Multiple clones were sequenced from each isolation. Sequences were submitted to GenBank under accessions MF373810 - MF373812.

New *Pseudotrichonympha* sequences were aligned with previously published sequences spanning the phylogenetic diversity of parabasalians using MAFFT with default settings^19^. Highly variable regions were removed using trimAl^20^ (settings: –gt 0.3 –st 0.001). Preliminary analyses confirmed with complete support that all new sequences corresponded to *Pseudotrichonympha*, and a more detailed analysis focused exclusively on sequences from this genus and outgroups, resulting in a final alignment of 28 taxa and 1,388 positions. ML analyses were performed with RAxML v8.2.9^21^, using the GTR + Γmodel and 1,000 random starting trees. For the ML analysis, support was assessed from 1,000 bootstrap replicates.

## Electronic supplementary material

Supplementary Information

Supplementary Video
