# Supplementary material for: Pseudotrichonympha leei, Pseudotrichonympha lifesoni, and Pseudotrichonympha pearti, new species of parabasalian flagellates and the description of a rotating subcellular structure
Source: Sci Rep. 2017 Nov 27;7:16349. doi: 10.1038/s41598-017-16259-8 (PMC5703945; doi:10.1038/s41598-017-16259-8)
Supplement: Supplementary file 1 — Supplementary Information [file 41598_2017_16259_MOESM1_ESM.pdf]

Pseudotrichonympha leei, Pseudotrichonympha lifesoni, and Pseudotrichonympha pearti, new species of parabasalian flagellates and the description of a rotating subcellular structure.

Javier del Campo[1,2], Erick R. James[1], Yoshihisa Hirakawa[1], Rebecca Fiorito[1], Martin Kolisko[1,3], Nicholas A. T. Irwin[1], Varsha Mathur[1], Vittorio Boscaro[1], Elisabeth Hehenberger[1], Anna Karnkowska[1,4], Rudolf H. Scheffrahn[5], Patrick J. Keeling[1,*]

[1] Department of Botany, University of British Columbia, Vancouver, British Columbia, Canada

[2] Department of Marine Biology and Oceanography, Institut de Ciències del Mar - CSIC, Barcelona, Catalonia, Spain

[3] Institute of Parasitology, Biology Centre, Czech Academy of Sciences, České Budějovice, Czech Republic

[4] Department of Molecular Phylogenetics and Evolution, Faculty of Biology and Biological and Chemical Research Centre, University of Warsaw, Warsaw, Poland

[5] Fort Lauderdale Research & Education Center, University of Florida, Davie, Florida, USA

[*]Corresponding author; pkeeling@mail.ubc.ca

Supplementary Figure Legends

Supplementary Figure 1. Barcode identification of termite hosts based on maximum likelihood phylogeny of mitochondrial 16S (LSU) rRNA. The tree is rooted with the closest known relatives to lower termites, *Cryptocercus punctulatus.* Numbers at nodes correspond to ML bootstrap support over 50% (nodes with lower support are not shown for clarity), and the scale bar represents a distance of 0.06 substitutions per site. Taxon names include GenBank accession numbers and the termite names. Sequences highlighted are *Coptotermes gestroi* and *Coptotermes cf. acinaciformis,* that were obtained in this study.

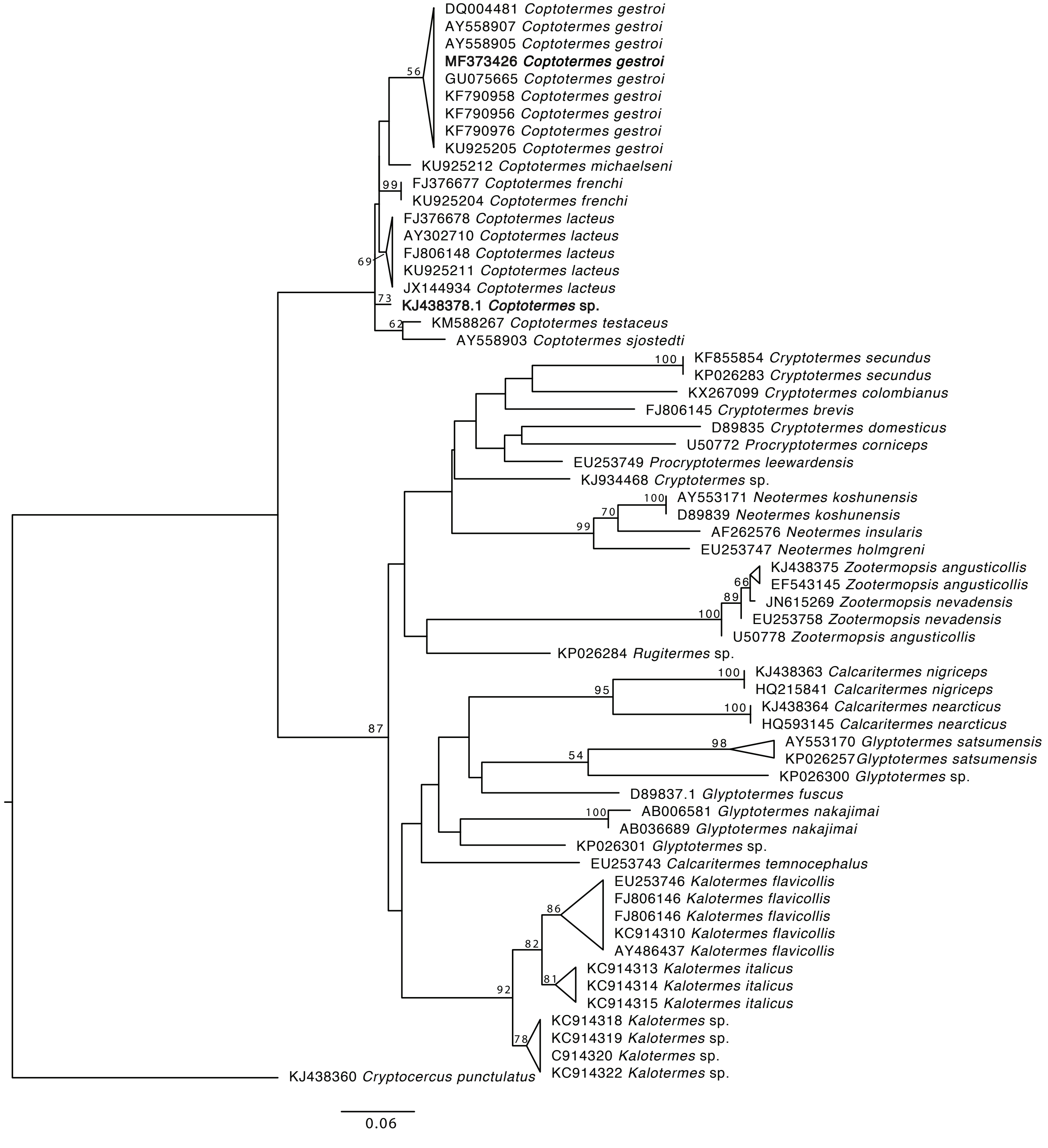

DQ004481 *Coptotermes gestroi*
AY558907 *Coptotermes gestroi*
AY558905 *Coptotermes gestroi*
MF373426 *Coptotermes gestroi*
GU075665 *Coptotermes gestroi*
KF790958 *Coptotermes gestroi*
KF790956 *Coptotermes gestroi*
KF790976 *Coptotermes gestroi*
KU925205 *Coptotermes gestroi*
KU925212 *Coptotermes michaelseni*
FJ376677 *Coptotermes frenchi*
KU925204 *Coptotermes frenchi*
FJ376678 *Coptotermes lacteus*
AY302710 *Coptotermes lacteus*
FJ806148 *Coptotermes lacteus*
KU925211 *Coptotermes lacteus*
JX144934 *Coptotermes lacteus*
KJ438378.1 *Coptotermes* sp.
KM588267 *Coptotermes testaceus*
AY558903 *Coptotermes sjostedti*
KF855854 *Cryptotermes secundus*
KP026283 *Cryptotermes secundus*
KX267099 *Cryptotermes colombianus*
FJ806145 *Cryptotermes brevis*
D89835 *Cryptotermes domesticus*
U50772 *Procryptotermes corniceps*
EU253749 *Procryptotermes leewardensis*
KJ934468 *Cryptotermes* sp.
AY553171 *Neotermes koshunensis*
D89839 *Neotermes koshunensis*
AF262576 *Neotermes insularis*
EU253747 *Neotermes holmgreni*
KJ438375 *Zootermopsis angusticollis*
EF543145 *Zootermopsis angusticollis*
JN615269 *Zootermopsis nevadensis*
EU253758 *Zootermopsis nevadensis*
U50778 *Zootermopsis angusticollis*
KP026284 *Rugitermes* sp.
KJ438363 *Calcaritermes nigriceps*
HQ215841 *Calcaritermes nigriceps*
KJ438364 *Calcaritermes nearcticus*
HQ593145 *Calcaritermes nearcticus*
AY553170 *Glyptotermes satsumensis*
KP026257 *Glyptotermes satsumensis*
KP026300 *Glyptotermes* sp.
D89837.1 *Glyptotermes fuscus*
AB006581 *Glyptotermes nakajimai*
AB036689 *Glyptotermes nakajimai*
KP026301 *Glyptotermes* sp.
EU253743 *Calcaritermes temnocephalus*
EU253746 *Kalotermes flavicollis*
FJ806146 *Kalotermes flavicollis*
FJ806146 *Kalotermes flavicollis*
KC914310 *Kalotermes flavicollis*
AY486437 *Kalotermes flavicollis*
KC914313 *Kalotermes italicus*
KC914314 *Kalotermes italicus*
KC914315 *Kalotermes italicus*
KC914318 *Kalotermes* sp.
KC914319 *Kalotermes* sp.
C914320 *Kalotermes* sp.
KC914322 *Kalotermes* sp.
KJ438360 *Cryptocercus punctulatus*
56
99
69
73
62
100
100
70
99
66
89
100
100
95
100
54
98
100
87
86
82
81
92
78
0.06
